# Diverse microbial metal resistance and novel metal cycling organisms in copper/nickel mine tailings

**DOI:** 10.1093/mtomcs/mfag028

**Published:** 2026-09-02

**Authors:** Molly Chen, Daniel S Grégoire, Jeffrey G Bain, David W Blowes, Laura A Hug

**Affiliations:** Department of Biology, University of Waterloo, Waterloo, Ontario, N2L 3G1, Canada; Department of Biology, University of Waterloo, Waterloo, Ontario, N2L 3G1, Canada; Department of Chemistry, Carleton University, Ottawa, Ontario, K1S 5B6, Canada; Department of Earth and Environmental Sciences, University of Waterloo, Waterloo, Ontario, N2L 3G1, Canada; Department of Earth and Environmental Sciences, University of Waterloo, Waterloo, Ontario, N2L 3G1, Canada; Department of Biology, University of Waterloo, Waterloo, Ontario, N2L 3G1, Canada

## Abstract

Mine tailings contribute to environmental heavy metal contamination through the formation of acid mine drainage. Microbially-mediated processes such as iron and sulfur redox cycling influence metal mobility. Here, we applied an integrated metagenomic and metaproteomic approach to profile microbial communities across vertical geochemical gradients in legacy copper/nickel tailings in Sudbury, Ontario, Canada. From 43 samples, we recovered 454 non-redundant metagenome-assembled genomes (MAGs), revealing diverse populations within the Actinobacteriota, Desulfobacterota, and uncultured lineages such as *Candidatus* Eremiobacterota and SZUA-79. Functional profiling identified 301 putative iron- and sulfur-cycling MAGs, including those within the *Ca*. Eremiobacterota and SZUA-79 phyla. A custom set of Hidden Markov Models (HMMs) was used to annotate metal resistance genes, which were widespread and diverse, but whose abundances did not correlate with measured Cu, Ni, or Fe concentrations. This observation suggests that resistance traits are broadly encoded in these microbial communities regardless of environmental metal concentrations. Proteomic data confirmed *in situ* expression of selected metal resistance genes and iron/sulfur metabolism genes, although protein recovery was limited due to the difficult nature of mine tailings as an extraction matrix. Our findings highlight both the depth of microbial diversity in metal resistance and metal biogeochemical cycling in mining waste, as well as the technical challenges that currently limit genomic and proteomic sequencing coverage in low-biomass, metal-rich matrices.

## Graphical Abstract

**Graphical Abstract:**
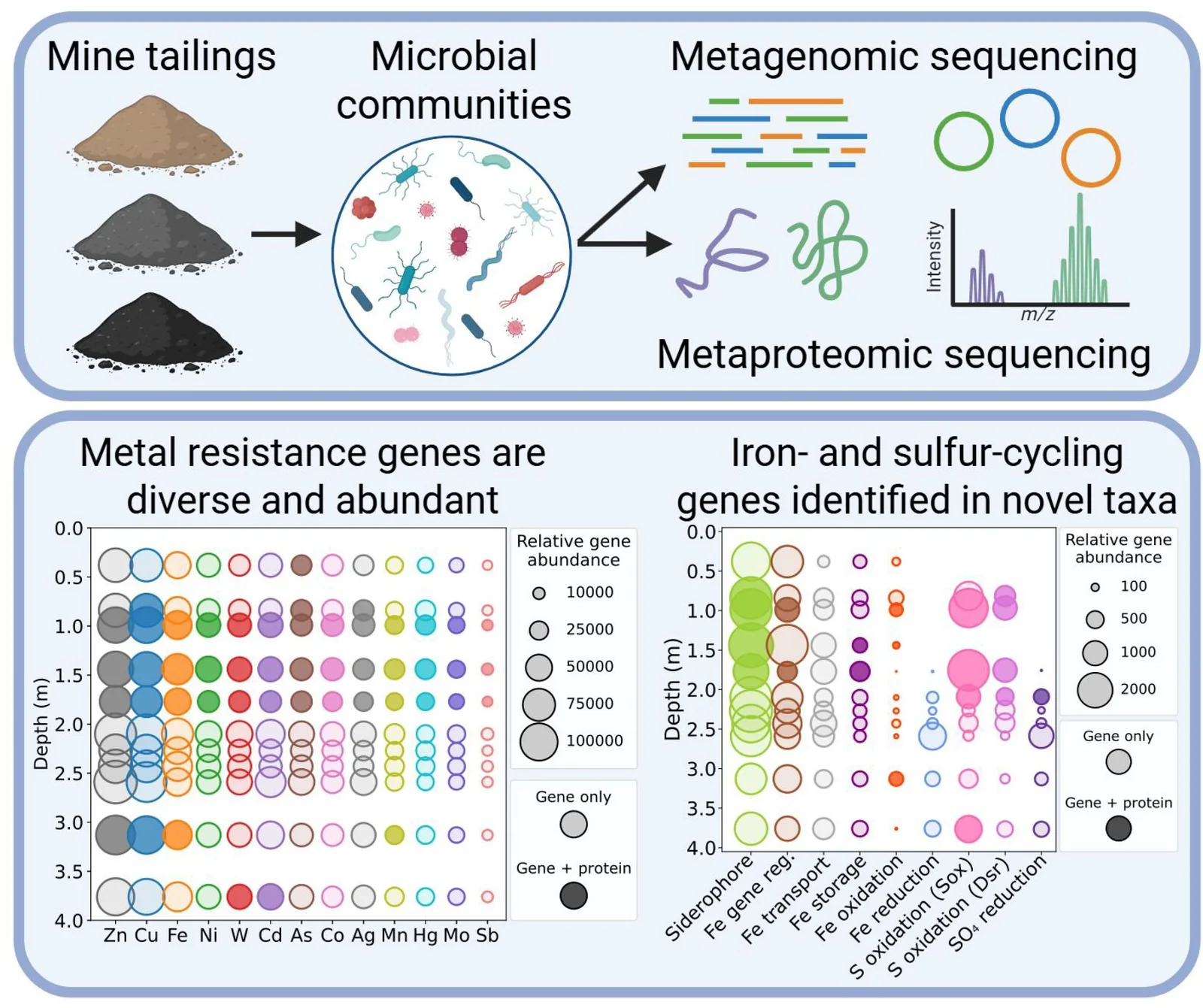
Mine tailings represent an environmental hazard due to the oxidation of sulfide minerals, releasing acid mine drainage and mobilizing heavy metals. Using multi-omics, we profiled microbial communities along vertical gradients in legacy copper/nickel tailings to predict microbial involvement in contaminant cycling. Metal resistance genes were widespread within metagenomes, and their abundances did not correlate with environmental metal concentrations. Iron- and sulfur-cycling were detected in many samples, including genes associated with previously unclassified and/or uncultured taxa.

## Introduction

Mine tailings are waste materials generated from the extraction of valuable metals from ores. After crushing ores into smaller particles and separating metals and minerals via flotation, density, and leaching [1], the residual fine-grained minerals and waste rock are typically mixed with water, forming a high-volume slurry of tailings that is transported to a designated storage facility [1].

Sulfide-rich tailings pose an on-going risk of environmental contamination due to the oxidation of sulfide minerals such as pyrite (FeS_2_), which leads to the formation of Acid Mine Drainage (AMD). AMD is characterized by moderately (pH < 7) to extremely (pH < 3) acidic wastewater with high concentrations of heavy metals [2]. Although mineral oxidation can occur abiotically, the activity of acidophilic chemolithoautotrophs (*e.g. Acidithiobacillus ferrooxidans, Leptospirillum ferrooxidans, Sulfolobus metallicus*) can significantly accelerate the rate of AMD formation by oxidizing ferrous iron (Fe^2+^) to ferric iron (Fe^3+^) and reduced inorganic sulfur compounds (RISCs) to sulfate (SO_4_^2−^) [2, 3]. These processes release AMD into soils and freshwater ecosystems, which is highly detrimental to environmental and human health (reviewed in [4]).

Mining waste microbial communities must possess adaptations to overcome the challenge of living in a harsh environment characterized by low pH and high concentrations of dissolved heavy metals. These adaptations rely on the use, acquisition, and/or evolutionary adaptation of metal resistance genes (MRGs). MRGs include membrane transporters for exporting metal ions outside of the cell [5], oxidoreductases for changing the redox state of metal ions into less toxic or bioavailable forms (*e.g*. from Cu^2+^ to Cu^1+^) [6], and metal-binding proteins for sequestering metal ions in the cytoplasm or periplasm [7]. Additionally, many microbes inhabiting tailings utilize iron- and sulfur-cycling genes in energy-generating metabolic pathways: these include iron- and sulfur oxidizing chemolithoautotrophs (examples above), as well as iron- and sulfate-reducing organisms (*e.g. Acidiphilium* spp., *Desulfovibrio* spp.) that use ferric iron and/or sulfate as alternative electron acceptors in anoxic tailings environments [2]; such genes will be referred to in this study as metal metabolism genes, or MMGs.

Understanding the microbial activities that control metal biogeochemical cycling in tailings is crucial for predicting the fate of metals in these systems and developing effective remediation strategies. For example, cover layers installed above tailings can restrict oxygen ingress, limit oxidation and facilitate anaerobic microbial metabolisms such as sulfate reduction, promoting secondary sulfide mineral precipitation [8]. To assess specific microbial metal-cycling activities, a comprehensive profile of microbial communities, including the MRG/MMGs they express, is required.

A previous investigation into tailings microbial communities identified high variability in community composition and diversity along depth gradients, and across four legacy copper/nickel tailings sites in Sudbury, Ontario [9]. The extent of sulfide mineral oxidation and presence of remedial cover layer(s) varied between sampling locations, influencing the relative acidity and dissolved metal concentrations of the environments. Through 16S rRNA amplicon sequencing on 10 cm vertical sections of tailings cores, we determined a high-resolution taxonomic profile of communities across a range of geochemical gradients. This study identified correlations between microbial community structures with pH, Eh, alkalinity, salinity, and some metal ions, although unexpectedly, Cu and Ni were not among the metal ions whose concentrations significantly correlated with community diversity. Many lineages associated with iron/sulfur cycling in tailings were identified, as well as a number of novel taxa that have not previously been described or studied within mine tailings [9].

Although 16S rRNA amplicon sequencing is a valuable tool for characterizing environmental microbial diversity, it does not allow for direct inference of the potential metabolic activities or functions of organisms. Predicting mechanisms of heavy metal resistance and biogeochemical cycling in mine tailings is improved by the use of metagenomics to identify MRGs and MMGs, especially those that are taxonomically novel or lack isolated representatives. Additionally, the activity and subsequent ecological impact of organisms in their natural environments are not necessarily tied to taxonomic or gene abundances. These roles can be clarified by measuring gene expression (*i.e*. via RNA and/or proteins) [10]. Metagenomic, metatranscriptomic, and metaproteomic (‘multi-omics’) studies of AMD environments have been crucial to advancing our knowledge of mining waste microbiology (reviewed in [11, 12]), providing information about the importance of rare populations [13], metal resistance strategies [14, 15], ecological succession [16], and potential for biomining [17, 18]. Metaproteomics even enabled the discovery of a novel cytochrome essential for iron oxidation within AMD communities [19].

It can be challenging to collect sequencing data from mine tailings due to the low biomass, high volume sample matrix, and the presence of heavy metal contaminants, which can interfere with processes such as DNA recovery and amplification [20]. Currently, there are no commercial products optimized for nucleic acid or protein extraction from tailings as there are for other types of microbiomes (*e.g*. soil, gut). Metagenomic studies on tailings communities have been conducted [21–23],  but metaproteomic studies of mining environments have focused on biofilms (reviewed in [24]), which are more feasible to collect from exposed surfaces in abandoned mine tunnels or surface water runoff channels. There have been no proteomic analyses of biomass extracted directly from mine tailings to date, likely due to lack of commercially available instrumentation and reagents specialized for and anticipated difficulties in extracting from these complex matrices.

The objective of this study was to combine metagenomic and metaproteomic approaches to identify microbial metal resistance mechanisms and iron/sulfur cycling capabilities in sulfidic copper/nickel mine tailings, located in two tailings areas in Sudbury, Ontario, Canada. To meet this objective, we optimized protein extraction from mine tailings, a challenging, low-biomass matrix. We used a combination of existing and custom-generated gene annotation tools to characterize MRG and MMGs (including sulfur-cycling genes) present in microorganisms inhabiting mine tailings. In doing so, we identified organisms of interest with high metal resistance and/or metal cycling potential, and considered how differences in MRG and MMGs between populations may be influenced by environmental variables such as metal concentrations.

## Methods

### Sampling and DNA extraction

Core sample collection and DNA extraction from mine tailings were performed as described in [9]. Briefly, cores measuring 5.0–7.6 cm (2–3 inches) in diameter and 3—5 m in length (depth below surface level) were extracted using a drill and aluminum pipe, capped, and transported to the lab for storage at −20°C prior to processing. Cores were sliced into 10 cm subsections, and 10 g of tailings from each subsample was weighed into a 50 ml falcon tube for DNA extraction using QIAGEN DNEasy PowerMax Soil kit. DNA samples were concentrated to a volume of 50–100 μl using ethanol/sodium acetate precipitation, and final DNA concentrations were determined using the Thermo Fisher Qubit dsDNA High Sensitivity Assay Kit. Samples were stored at −20°C prior to sequencing.

### Geochemical assessments

Pore-water samplers (Soil Moisture Inc. model 1900 soil water solution samplers) were installed at each location at 10–20 cm depth intervals at ML25 and ML34 sampling locations, and water was collected from samplers after approximately one week. Piezometers were used to collect pore-water at NR3 (0.15–0.4 m intervals), as well as at NR18 below depths of 1.13 m. Pore-water pH, Eh, alkalinity, and electrical conductivity were measured on-site. Remaining water samples were filtered (0.45 µm) into 20 ml bottles and stored at 4°C for further analysis: inductively coupled plasma-optical emission spectrometry and inductively coupled plasma-mass spectrometry (ICP-OES/ICP-MS) for cation concentrations, ion chromatography (IC) for anion concentrations, and non-dispersive infrared sensor (NDIR) analysis for total dissolved organic carbon (DOC).

### Metagenomic sequencing, assembly, and binning

A total of 112 DNA samples were obtained. Of these, 43 samples were sent for metagenomic sequencing based on 16S rRNA sequencing results [9]. Selection criteria aimed to maximize the range of geochemistry and microbial community compositions observed across samples, while minimizing redundancy. For example, if a group of adjacent samples in one core had similar concentrations of dissolved metals, pH, and relative taxon abundances, one representative sample from the group was selected for metagenomic sequencing.

Paired-end metagenomic sequencing was performed at The Center for Applied Genomics (TCAG) (Toronto, Ontario), with an Illumina NovaSeq SP flowcell (2 × 150 bp). Libraries were PCR-amplified prior to sequencing. Raw reads were decontaminated with BBduk [25], trimmed/quality filtered using sickle v1.33 [26]. SPAdes v3.15.5 [27] was used to assemble reads, with the—meta flag for metagenomes.

For binning, a set of assembled scaffolds ≥ 2500 bp were filtered using pullseq v1.0.2 [28]. Bowtie2 v2.3.4.1 [29] was used for read mapping based on sequencing depth to generate scaffold coverage information. Scaffolds were independently binned with three binning algorithms: CONCOCT [30], MetaBAT 2 [31], and MaxBin 2.0 [32], after which the dereplication, aggregation, and scoring tool (DAS Tool) [33] was used to select the best set of high-quality non-redundant bins from the candidate bins. CheckM [34] was used to filter high-quality bins based on >70% completeness and <10% contamination thresholds.

After binning, MAGs were dereplicated across all samples with dRep [35] to generate a non-redundant set of MAGs for gene annotation.

### Metagenome annotation and data analysis

MAG taxonomy was assigned with GTDB-tk (v2.1.0, database release version 207.0) [36]. Phylogenetic trees were constructed with GToTree [37], and visualized with the online interactive tree of life (iToL) tool [38]. Iron-related genes were annotated with FeGenie [39], and sulfur metabolism genes were taken from DRAM (Distilled and Refined Annotation of Metabolism) annotations [40]. DiSCo [41] was also used for sulfur gene annotation, which can distinguish between oxidative and reductive variants of *dsr* genes.

MRG gene annotation was based on the BacMet (v2.0) antibacterial biocide- and metal-resistance genes database [42]. The BacMet 2.0 database contains 753 curated, experimentally verified bacterial metal and biocide resistance genes, as well as 155 512 predicted genes based on sequence similarity to the experimentally verified database [42]. Initially, MRGs were annotated with the BacMet-scan software provided with the database, which uses a Basic Local Alignment Search Tool (BLAST) algorithm to identify similar genes. Prodigal [43] was used to predict open reading frames (ORFs) from MAGs for BacMet-scan, and results were filtered using a E < 0.05 threshold.

The BacMet-scan method resulted in limited hits, so we developed an alternative method of MRG identification that would be comparable to the other gene annotation tools used in this study (FeGenie, DRAM, DiSCo) based on creating profile HMMs for MRGs of interest. Multiple sequence alignments (MSAs) were generated with MUSCLE (v3.8.31) [44] for protein sequences associated with each of the 613 unique genes present in the combined BacMet experimentally verified and predicted gene databases. MSAs were verified by visual inspection in Geneious (v5.6) [45]. Profile HMMs were generated from MSAs using HMMER (v3.2.1) [46], excluding genes with <5 representative sequences, resulting in 529 HMMs The set of predicted ORFs used in the initial BacMet-scan were screened with the developed BacMet HMMs using HMMER, with E < 0.05 indicating a significant hit.

To track the distribution of MRGs in MAGs compared to unbinned scaffolds, including mobile genetic elements, BacMet HMM hits for all scaffolds ≥2.5 kbp were aggregated and mapped to each scaffold’s classification from PPRmeta [47].

All data from FeGenie, DRAM, DiSCo, BacMet-scan, and BacMet HMM searches were exported to Python for analysis using the pandas library [48]. Gene frequencies were aggregated into categories (*e.g*. iron oxidation, sulfur reduction, copper resistance) and normalized to MAG relative abundance as a proxy for relative gene abundance, which was calculated from the average scaffold coverage of each MAG divided by the total coverage of all binned scaffolds within a sample. Figures were generated using matplotlib [49].

### Protein extraction and sequencing

The full set of samples selected for metagenomics were also subjected to metaproteomic sequencing.

The method of protein extraction was modified from a protocol described in Keiblinger et al. [50], which compared the effectiveness of multiple protein extraction protocols on soil samples and found that an SDS-phenol extraction method [51] resulted in the highest numbers of mapped proteins from LC—MS/MS [50]. Total 10 g of tailings were measured into 50 ml falcon tubes and pre-washed with 10 mM Tris-EDTA buffer. The following was then added to sample tubes: 5 ml each of stainless steel and garnet beads, 15 ml of 1% SDS in 50 mM Tris-HCl pH 8.0, and 15 ml of Tris-HCl equilibrated liquid phenol. Samples were vortexed for 10 min and sonicated with a probe sonicator for 30 s, with all samples stored on ice between steps. Tubes were then placed onto a horizontal shaker at 160 rpm for 1 h at room temperature, after which the vortex and sonication steps were repeated. Samples were centrifuged for 20 min at 3500 × g at 4°C to separate the phenol layer (containing the solubilized proteins) from the aqueous layer. The phenol phase was transferred into a new 50 ml tube for precipitation of proteins overnight in 5 volumes of 0.1 M ammonium acetate in methanol at −20°C.

To prepare peptides for LC-MS/MS, proteins were centrifuged for 20 min at 10 000 x g at 4°C, washed with 2 ml of cold acetone, and resolubilized in 300 µl of denaturation buffer (8 M urea in 50 mM Tris-HCl). Samples were then incubated with 10 mM dithiothreitol (DTT) at 60°C for 30 mins to reduce disulfide bonds, then with 20 mM iodoacetamide (IAA) at room temperature for 30 mins to alkylate cysteines. Proteins were precipitated again in acetone, dried, and resuspended in 50 mM ammonium bicarbonate for overnight digestion with 1.5 µg trypsin at 37°C. The digest was acidified with 1% formic acid, after which peptides were purified using Pierce C18 spin columns (Thermo Fisher #89 870), dried in a vacuum centrifuge, and stored at −80°C prior to LC—MS/MS.

LC-MS/MS was performed in the Biomolecular Core (BioCORE) Facility at Western University Schulich School of Medicine and Dentistry, London, Ontario, Canada, with the following specifications: peptides were resuspended in 1% acetonitrile (ACN) and 0.1% formic acid (FA), placed in an ultrasonic bath for 20 min, and centrifuged at 21 000 × g for 7 min at room temperature. Peptide samples were injected onto a Waters Acquity Ultra-Performance Liquid Chromatography (UPLC) M-Class system (Waters, Milford, MA) coupled to a Q Exactive Plus hybrid quadrupole-Orbitrap mass spectrometer (Thermo Fisher Scientific, Waltham, MA). Mobile Phase A consisted of water/0.1% FA and Mobile Phase B consisted of ACN/0.1% FA. Samples were trapped for 5 min at a flow rate of 5 μl/min using 99% Buffer A, 1% Buffer B on a Symmetry C18 Trap Column, 5 μm, 180 μm × 20 mm (Waters). Peptides were separated using a Peptide BEH C18 Column, 130 Å, 1.7 μm, 75 μm × 250 mm operating at a flow rate of 300 nl/min at 35°C (Waters). Samples were separated using a nonlinear gradient consisting of 1%–7% B over 1 min, 7%–23% B over 135 min and 23%–35% B over 180 min before increasing to 99% B, for a total time of 3 h. The Q Exactive Plus mass spectrometer was controlled by Thermo Xcalibur software (v4.0). Data were acquired in the data-dependent acquisition mode (DDA) using a Fourier Transform/Higher-Energy Collision Dissociation (FT/FT/HCD) Top 12 scheme. MS1 survey scans were performed from 375 to 1500 m/z at a resolution of 70 000 with Automatic Gain Control (AGC) set to 3e6 and a maximum injection time of 250 ms. Multiply charged peptide ions were isolated using the quadrupole with an isolation window of 1.2 m/z and were fragmented using HCD with Normalized Collision Energy (NCE) set to 25%. MS2 scans were performed at a resolution of 17 500 with AGC set to 2e5 and a maximum injection time of 64 ms. Dynamic exclusion was set to 40 s, and lock mass ion was enabled at 445.120 025 m/z.

### Metaproteome annotation and data analysis

Initial data processing was conducted by personnel at the BioCORE facility, using the PEAKS 12 software (Bioinformatics Solutions Inc., Waterloo, Canada). To map predicted proteins, a custom ORF database was generated from the metagenomic sequencing data from this study by using Prodigal to predict ORFs in assembled scaffolds above 1 kbp in length. Predicted proteins from all metagenomes were combined into a single database to search the MS data against, with the following specifications: a precursor ion mass tolerance was set to 10 ppm, and a fragment ion mass tolerance was set to 0.02 Da. Specific cleavage with trypsin was selected with a maximum of two missed cleavages. A fixed modification of carbamidomethylation and variable modifications of deamidation, oxidation and acetylation were added, and a maximum of two variable modifications were allowed per peptide. The peptide false discovery rate (FDR) was set to 1% for the database search. One unique peptide and an FDR of less than 1% were applied for confident protein identification.

Comparison of protein abundances across samples was not possible as all extracted peptides from each sample were injected to maximize the signal detection potential for low biomass samples, and therefore the amount of peptide injected varied between samples. Relative abundances within a sample were also not compared as the method of data collection did not account for differences in peptide detection ability by mass spectrometry. As a result, analysis of proteomic data was limited to inferring microbial activity based on the positive identification of associated proteins.

## Results and discussion

### Sampling site characteristics

Sampling occurred in November 2021 from four sampling locations at two sulfide tailing impoundments operated by Glencore Sudbury Integrated Nickel Operations (Sudbury INO): ML25 and ML34, from the Strathcona Tailings Management Area, which received tailings from 1968 to 2012; as well as NR18 and NR3, from the Nickel Rim North tailings area, which received tailings from 1953 to 1958 [9, 52].

ML25 tailings are highly oxidized and pore-water metal concentrations are relatively high compared to ML34, NR18, and NR3. Tailings near ML34 are overlain by an organic cover layer (biosolids and municipal compost) that was around 0.4 m thick at the time of sampling, limiting oxidation of the underlying sulfide minerals, which was included in the sample material. Additional details on mineral composition, storage conditions, and contaminant profiles for each of the sampling locations are described here when relevant, and more fully in [9].

### Sequencing statistics

A total of 757 high-quality (>70% complete, <10% contaminated) metagenome-assembled genomes (MAGs) (454 non-redundant, after dereplication) were identified across 43 samples (ranging from 1–54 per sample, average of 18) after assembly and binning of metagenomic sequencing data (Table 1). The number of high-quality MAGs per sample was much lower than the number of ASVs identified through 16S rRNA amplicon sequencing (minimum ASVs per sample = 141) [9]. One sample (ML25_013) did not return any high-quality bins and was excluded from subsequent analyses involving MAG gene annotation. Low MAG counts were often associated with a low fraction of assembled reads (Table 1), likely resulting from insufficient sequencing coverage of low biomass samples, despite PCR amplification during the metagenome library preparation step.

**Table 1.** Metagenomic sequencing statistics by sample.

Sample	Depth (m)	Reads	% Assembled	Scaffolds	Scaffolds binned	HQ MAGs
ML25_003	0.28	16 647 582	76.03	15 966	8387	33
ML25_006	0.62	21 542 193	79.92	18 432	7081	32
ML25_007	0.73	13 168 935	84.90	8715	4309	22
ML25_008	0.84	16 652 969	89.41	8187	3727	19
ML25_009	0.96	36 061 742	95.62	6644	3579	24
ML25_010	1.07	37 476 942	89.22	4672	2323	13
ML25_012	1.29	3 838 337	30.03	1985	679	2
ML25_013	1.42	4 011 559	0.59	7	0	0
ML25_014	1.59	37 774 736	75.19	28 541	12 878	48
ML25_015	1.77	13 085 404	38.51	3274	1536	7
ML25_016	1.94	40 345 890	77.70	15 366	6124	28
ML25_022	3.03	33 927 710	73.12	13 490	3550	16
ML25_026	3.87	4 246 725	62.65	958	585	4
ML34_003	0.38	18 535 314	15.74	11 019	1458	5
ML34_006	0.84	19 429 820	73.10	16 192	5748	26
ML34_007	0.99	20 155 676	80.16	13 528	5123	25
ML34_010	1.44	20 207 295	3.85	3718	1187	3
ML34_012	1.77	28 980 246	94.50	3862	2362	15
ML34_014	2.10	19 268 023	79.96	13 333	3868	22
ML34_015	2.27	26 572 506	80.72	17 751	7788	45
ML34_016	2.43	44 069 643	75.03	31 683	12 236	54
ML34_017	2.59	39 831 849	83.75	13 007	5360	22
ML34_020	3.13	21 090 603	62.35	26 331	9575	36
ML34_022	3.76	16 805 500	70.14	15 098	4554	21
NR18_003	0.25	17 734 958	10.25	12 762	872	3
NR18_005	0.45	23 358 319	73.17	18 185	6182	38
NR18_007	0.65	18 924 563	72.48	16 652	6220	29
NR18_010	0.95	18 483 671	70.87	15 895	7177	33
NR18_011	1.05	16 063 433	73.97	14 261	4992	28
NR18_012	1.15	18 525 675	84.53	12 613	5359	28
NR18_015	1.51	18 780 223	4.32	5533	214	1
NR18_017	1.86	21 590 172	84.44	2485	1306	7
NR18_023	2.92	35 560 955	14.00	15 853	2880	12
NR18_025	3.27	45 504 942	85.73	2969	1443	6
NR18_028	3.76	12 877 162	16.17	1692	410	2
NR3_003	0.34	5 781 590	51.47	740	497	4
NR3_006	0.73	40 278 890	95.24	2983	1606	7
NR3_009	1.11	7 729 451	67.07	582	361	3
NR3_012	1.57	8 307 886	33.98	458	458	2
NR3_017	2.72	2 971 431	10.36	794	16	1
NR3_023	3.11	20 759 186	33.60	12 394	4172	21
NR3_025	3.37	45 504 942	11.75	1388	751	2
NR3_028	3.83	12 877 162	17.65	11 726	1735	8
Total	N/A	927 847 495	N/A	441 724	160 695	757

Scaffolds were binned into metagenome-assembled genomes (MAGs) using MetaBAT2, CONCOCT, and MaxBin2, and DAS Tool was used to identify the highest quality set of non-redundant bins among the three binning algorithms. CheckM was used to filter for bins with >70% completeness and <10% contamination as the final set of high-quality (HQ) MAGs. Samples are named by site (ML, NR), core number, and then 10 cm depth sample number (*e.g*. ML25_006 and ML25_007 are from the same site and core, and are sequential depth samples starting at 60 and 70 cm deep, respectively).

Positive identification of proteins from LC-MS/MS was only possible for 27 out of 43 submitted protein samples. A total of 29 069 proteins were identified (ranging from 1–8192 per sample, average of 1077) (Table 2), the majority of which were unique (24 033). Only 10 666 proteins (37%) mapped to binned scaffolds, with the rest mapping to proteins predicted from unbinned scaffolds. A major issue impacting data collection was the presence of polyethylene glycol (PEG), a common polymer contaminant in mass spectrometry-based analyses [53], which presents as numerous peaks with masses increasing sequentially by 44 kDa, swamping true biological signals [54]. Avoiding PEG contamination is challenging, as it is ubiquitous in many commercial and laboratory products, such as cosmetics, detergents and plastics [54]. Attempts to limit contamination during protein extraction by using HPLC-grade solvents, rinsing glassware with isopropanol instead of soap, and avoiding the use of PEG-containing plastics led to marginal improvements in some samples, but significant PEG contamination was still detected in others. It is possible that PEG was introduced to samples from handling prior to protein extraction, such as during tailings core processing. Additional processing with detergent removal and desalting columns were performed at the BioCORE facility (University of Western Ontario, Canada, see Methods), but these steps did not result in an improvement on the observed relative peptide signal.

**Table 2.** Metaproteomic sequencing statistics by sample.

Sample	Depth (m)	Protein #	Peptide #	Unique peptides
ML25_003	0.28	8	8	4
ML25_006	0.62	8192	20 044	6796
ML25_007	0.73	2065	2866	801
ML25_009	0.96	737	759	660
ML25_010	1.07	532	537	445
ML25_013	1.42	6	6	4
ML34_003	0.38	179	193	66
ML34_006	0.84	291	403	84
ML34_007	0.99	2036	3833	1888
ML34_010	1.44	2770	3964	1024
ML34_012	1.77	5470	11 675	3863
ML34_014	2.1	9	9	7
ML34_015	2.27	61	68	17
ML34_016	2.43	20	22	7
ML34_017	2.59	1	2	2
ML34_020	3.13	365	550	252
ML34_022	3.76	47	52	12
NR18_003	0.25	58	62	32
NR18_010	0.95	204	286	69
NR18_011	1.05	734	979	250
NR18_012	1.15	65	70	7
NR18_015	1.51	206	208	46
NR18_023	2.92	585	591	464
NR18_025	3.27	2	2	2
NR3_009	1.11	489	494	418
NR3_023	3.11	548	558	491
NR3_028	3.83	3389	5069	2342
Total	N/A	29 069	53 309	20 053

Peptides were identified using LC-MS/MS and mapped to a non-redundant database of predicted proteins from assembled scaffolds >1 kb from this study, with proteins clustered at 99% ID and minimum 80% length of the longest protein in a cluster. The total number of expressed proteins within a sample is reported as the Protein # along with the total number of Peptides supporting the identified proteins (Peptide #).  Peptides may be shared among multiple proteins. Unique peptides are defined as peptides that map to a single protein group (which may include redundancies due to homologs and variable isoforms not clustered during our database creation due to length or sequence identity variation). A minimum of one unique peptide within a protein group and a false-discovery rate of 1% was used as the threshold for positive protein identification.

The relatively small number of MAGs, even for a low-diversity community, alongside the low number of identified proteins in this study reflect the ongoing challenges in obtaining high-quality DNA and protein samples from mine tailings. At baseline, mine tailings exhibit many of the same issues as soil for DNA and protein extractions, including the presence of high molecular weight humic acids and recalcitrant clays [55]. Further, low biomass and contaminants within the sample matrix (*e.g*. heavy metals) limit the effectiveness of current DNA/protein extraction methods, which are optimized for soil communities. Low biomass protein samples will also be sensitive to contaminants introduced during sample processing (*e.g*. PEG), resulting in low signal-to-noise ratios in mass spectrometry. Nevertheless, these paired metagenome/metaproteome samples allowed for examination of the abundant members of the tailings microbial communities, and, for some samples, a robust determination of highly expressed genes via proteomics. Though less comprehensive than initially hoped, this still represents a unique dataset of DNA and protein extracted directly from mine tailings.

### Microbial community overview

**Figure 1.**
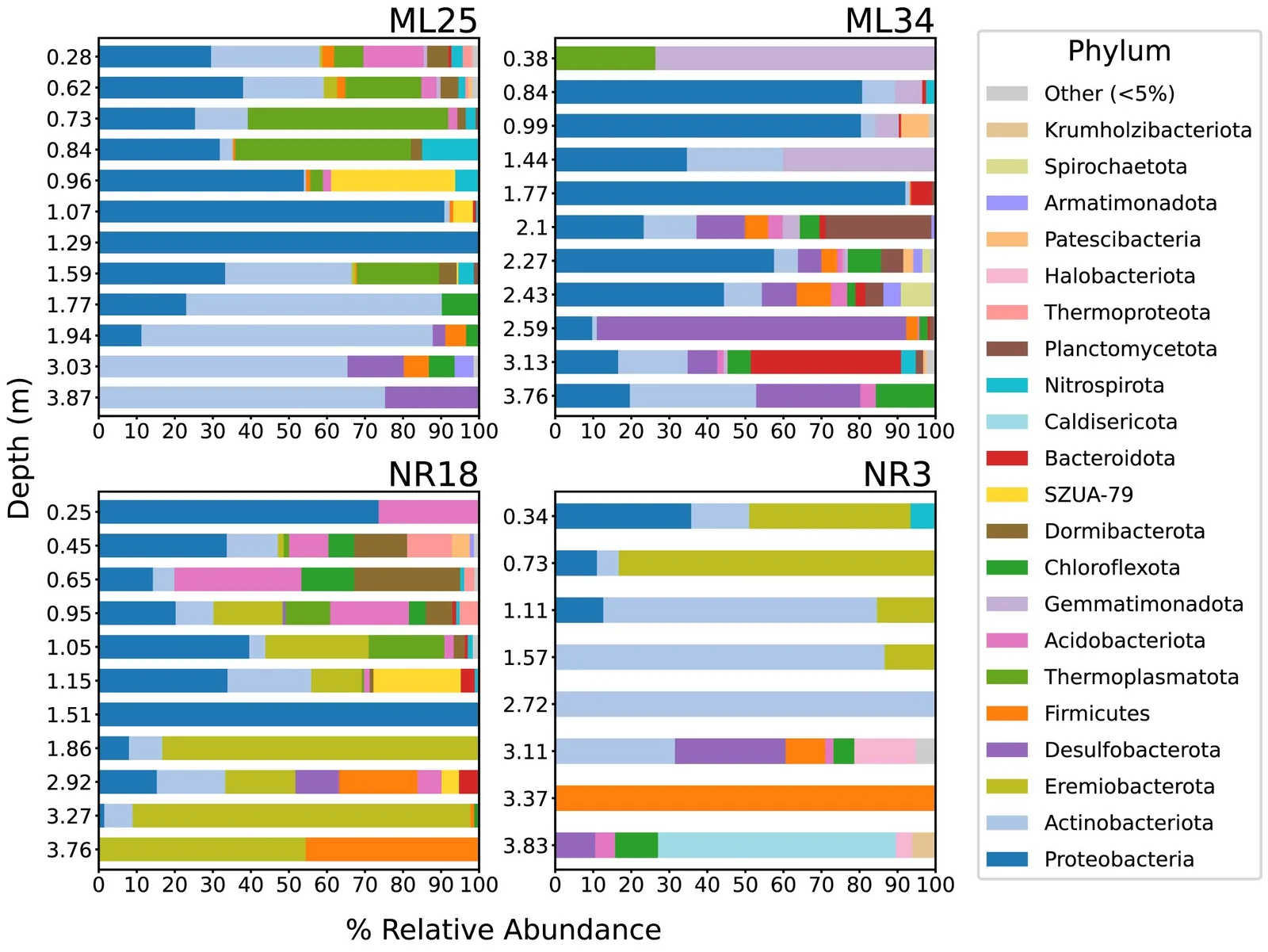
Microbial community composition across tailings cores from metagenomic sequencing data. Bars indicate phylum-level relative abundances of MAGs within each sample, based on the average depth of coverage of scaffolds within individual MAGs. Only phyla present at a relative abundance >5% in at least one sample in any dataset are shown. Phyla are ordered from most overall abundant (left) to least abundant (right), or bottom-to-top in the legend. MAGs were classified with GTDB-tk (v2.1.0, database release version 207.0). Note that nomenclature for the Proteobacteria (Pseudomonadota) and Firmicutes (Bacillota) have been updated in more recent versions of the GTDB. The y axes indicate the sample depth below ground level and are not scaled proportionally.

Community profiling based on metagenomic sequencing data was low resolution due to the limited number of high-quality MAGs. In some cases, a sample appeared to be comprised of only 1–2 populations (Fig. 1), but it was clear from 16S rRNA amplicon sequencing [9] that not all of the community diversity was captured in the assembled and binned scaffolds, especially for lower-abundance organisms. Consequently, quantitative comparisons of relative MAG abundances in this study (Fig. 1) should be viewed with caution, especially for samples with low bin counts (<10). However, the presence and localization of abundant taxa (>15%) generally agreed between the two datasets (Fig. 1) [9].

**Figure 2.**
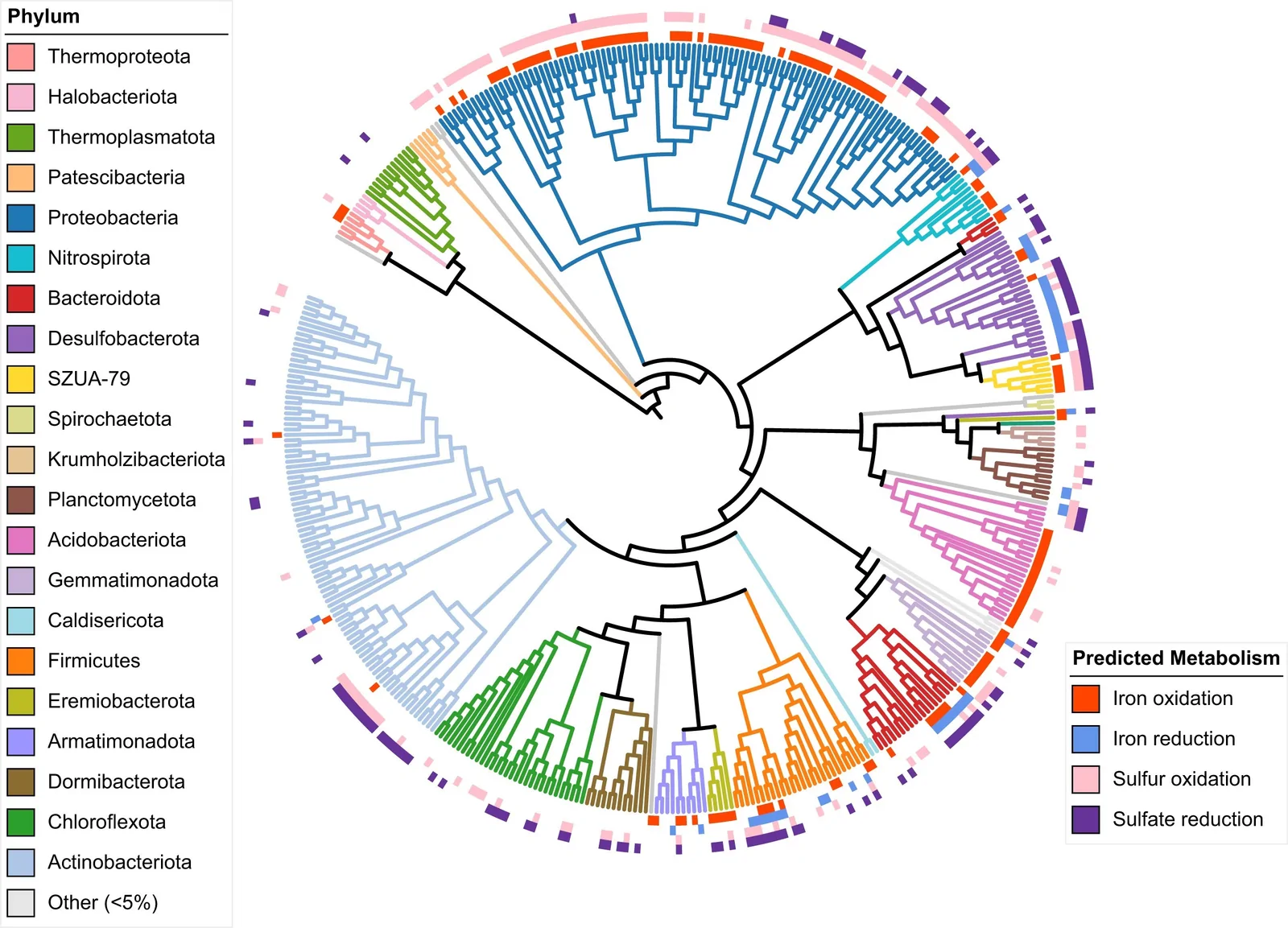
Phylogenetic distribution of sulfur and iron cycling genes across all MAGs. Tree branches are unscaled (cladogram) and colored by GTDB-tk assigned phylum in clockwise order following the legend, starting from the archaea (top left). Only phyla present at a relative abundance >5% in at least one sample in any dataset are colored. Outer rings represent presence/absence of iron and sulfur cycling genes, where at least one hit for any gene within each category was denoted as presence. Iron and sulfur metabolism genes were compiled from FeGenie, DRAM, and DiSCo annotations. Iron oxidation genes include *cyc1*/*cyc2, fox* genes, and sulfocyanin; reduction genes include *omc* and *dfe* genes. Sulfur oxidation is represented by genes in the *sox* or oxidative type *dsr* pathways; reduction genes include thiosulfate reductase, tetrathionate reductase, and reductive type *dsr* genes.

In terms of phylogenetic diversity, the Proteobacteria (mainly Gammaproteobacteria) and Actinobacteriota phyla had the highest overall abundance (Fig. 1) and the highest number of non-redundant MAGs across samples (Fig. 2). Most of the identified proteobacterial MAGs belong to clades associated with iron and/or sulfur oxidation in mining waste, but there were differences in the localization of individual taxa. Within the Acidiferrobacterales, *Acidiferrobacter* spp. were unique to ML25 whereas *Sulfuricaulis* spp. were almost exclusively found in NR18. *Sulfuricella, Sulfuriferula*, and *Thiobacillus* spp. (o. Burkholderiales) were mainly found in ML25 and ML34, with *Thiobacillus* much more abundant in ML34 samples. *Acidithiobacillus* spp. were found in all four tailings locations, but *A. ferrooxidans* were highly abundant in ML25, whereas *A. ferrivorans* was more common in other samples.

Unlike the Proteobacteria, Actinobacteriota MAGs were relatively novel, with most unclassified at the family or genus level, and with only one mining waste-associated Actinobacteriota (*Ferrimicrobium* spp.) identified, and only at <4% abundance. The majority of Actinobacteriota in shallow samples (<2 m) belong to the Acidimicrobiales order, and appear to be mostly organoheterotrophs. Deeper samples (>2 m) included members of the understudied anaerobic Thermoleophilia and Humimicrobiia clades, some of which are predicted to be sulfur oxidizers and/or sulfate reducers based on *sox* and/or *dsr* gene annotations (Fig. 2).

Other abundant lineages mapping to predicted roles in iron and/or sulfur cycling included the Desulfobacterota, Acidobacteriota (ML25/NR18), Bacteroidota (ML34), Firmicutes (NR18/NR3), SZUA-79 (ML25/NR18), and Eremiobacterota (NR18/NR3), which are discussed in that context below. Abundant phyla that did not contribute to the observed iron/sulfur cycling gene abundances or gene expression included the archaea (Thermoplasmatota), Caldisericota, Planctomycetota, Gemmatimonadota, and Dormibacterota. For a detailed description of the microbial community taxonomic correlations with overall geochemistry across the site and along core depths, see the work presented in (Chen et al. 2024).

### Distribution and expression of MRGs

Annotating MRGs using the built in BacMet-scan software associated with the BacMet bacterial metal and biocide resistance gene database [42] returned 329 520 non-redundant hits from all binned scaffolds. Annotation using custom Hidden Markov Models (HMMs) built from the same BacMet database (see Methods) returned 1 660 455 hits at the same level of statistical significance (E < 0.05) (Table S1). The BLAST-based approach used by BacMet-scan is generally considered less sensitive than HMMs [56, 57], where HMMs can identify more evolutionarily distant homologs across gene families, enabling the detection of a broader range of genes [57]. The disadvantage to an HMM-based approach is that more divergent genes have a higher likelihood of spurious annotations, reducing confidence in the predicted functions. On balance, the advantages of HMMs outweigh the disadvantages for samples from relatively poorly characterized environments, and so we proceeded with data analysis using results from our HMM-based gene annotations. The HMM set excluded genes with < 5 representative sequences in the BacMet database (84 out of 613 unique genes). To partially account for this, the BacMet-scan BLAST hits for these 84 genes were appended to the HMM annotations, which added 10 462 additional hits, or a 0.6% increase. Both methods lack the ability to annotate archaeal genomes as the BacMet database only includes bacterial sequences. This means the 22 archaeal MAGs of the 454 total non-redundant MAGs (4.8%) were not included in this analysis.

**Figure 3.**
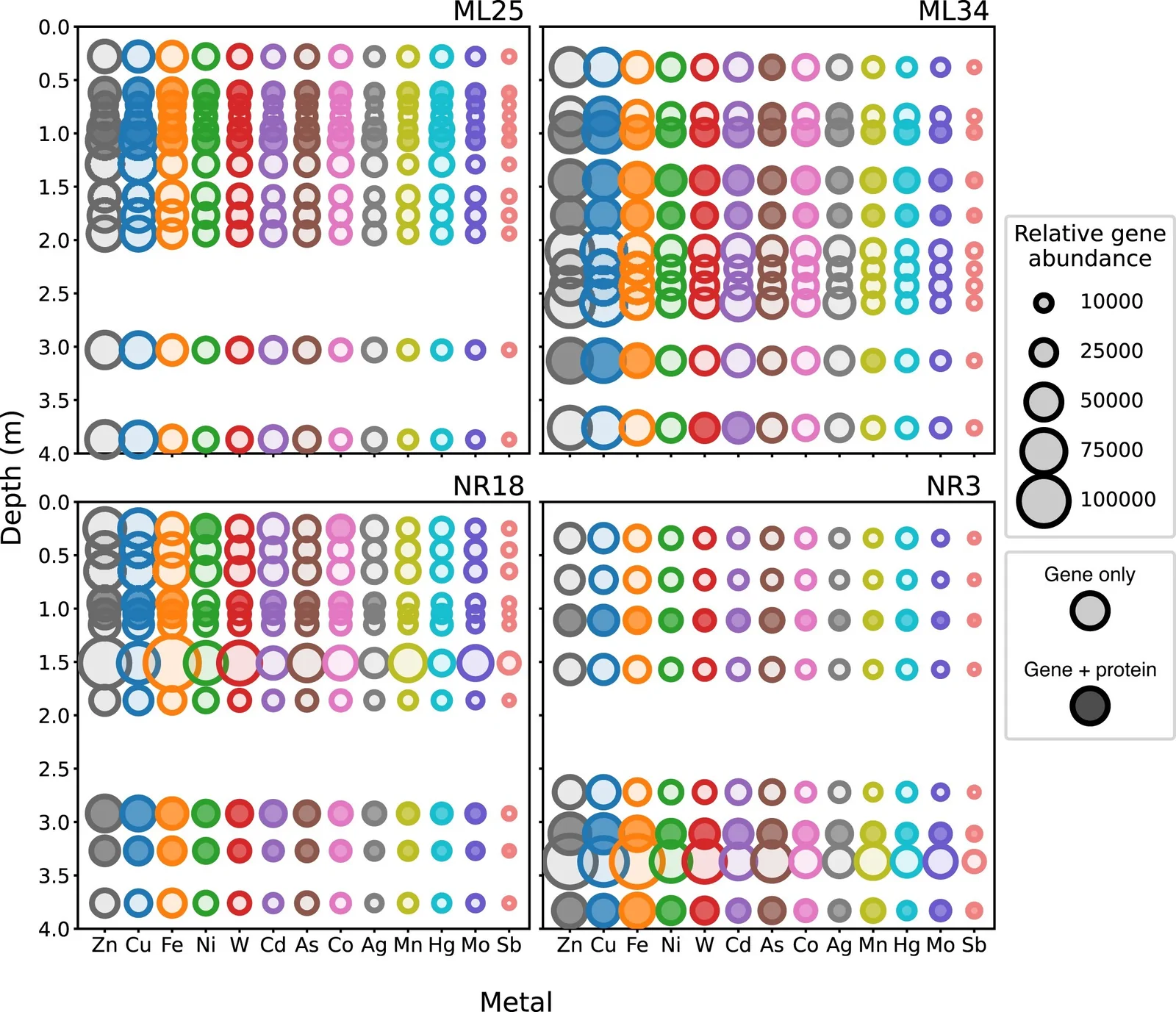
Distribution of predicted MRGs across vertical mine tailings transects. MAGs were annotated using profile HMMs of genes in the BacMet database. Rare genes (<5 representatives in the BacMet database) were annotated with BacMet-scan. Circle diameters representing relative gene abundances were calculated as the number of hits multiplied by the percent relative abundance of the MAG associated with each hit. In samples where protein expression was detected, the corresponding gene(s) are indicated by circles with darker shading. MRGs are colored based on metal, ordered from highest overall abundance (Zn) on the left to lowest (Sb) on the right. Multi-metal resistance genes were counted towards multiple categories. Only categories with abundances >2% of the total are shown.

The overall abundance of our predicted MRGs was generally consistent across samples within each tailings core, with two exceptions in NR18 (1.51 m) and NR3 (3.37 m), both showing elevated proportions across MRGs (Fig. 3). These samples had poor assembly and binning statistics, with only three total high-quality MAGs, (Table 1) and thus MRGs predicted from these MAGs are likely over-represented compared to the complete microbial communities. When comparing gene abundances by metal, zinc MRGs were the most common, followed by copper, nickel, and iron (Fig. 3). Database biases may contribute to these observed MRG abundances, as the highest number of metal resistance genes in the BacMet database are attributed to copper (60), zinc (58), and nickel (51) [42]. Most MRGs associated with zinc exhibit broad specificity and also confer resistance to other metals (most commonly cadmium and copper) [42], which could explain the overrepresentation of zinc MRGs, as these MRG hits would be counted multiple times, despite zinc not being a major contaminant in these tailings (<9.0 mg/l) compared to the levels of iron, nickel, and copper at these sites (Supplemental Data File 1: Table S2).

Differences in metal toxicity may also affect the relative abundance of their associated MRGs. Resistance mechanisms for highly toxic metals such as arsenic and mercury are likely to be encoded in microbial genomes regardless of whether environmental concentrations are high, as these pathways evolved early in Earth’s history and are widely distributed among bacteria and archaea [58, 59]. Likewise, copper resistance genes were more abundant than nickel and iron resistance genes (Fig. 3), even though copper concentrations were lower in most samples, likely reflecting copper’s greater cytotoxicity relative to iron and nickel [60, 61].

**Figure 4.**
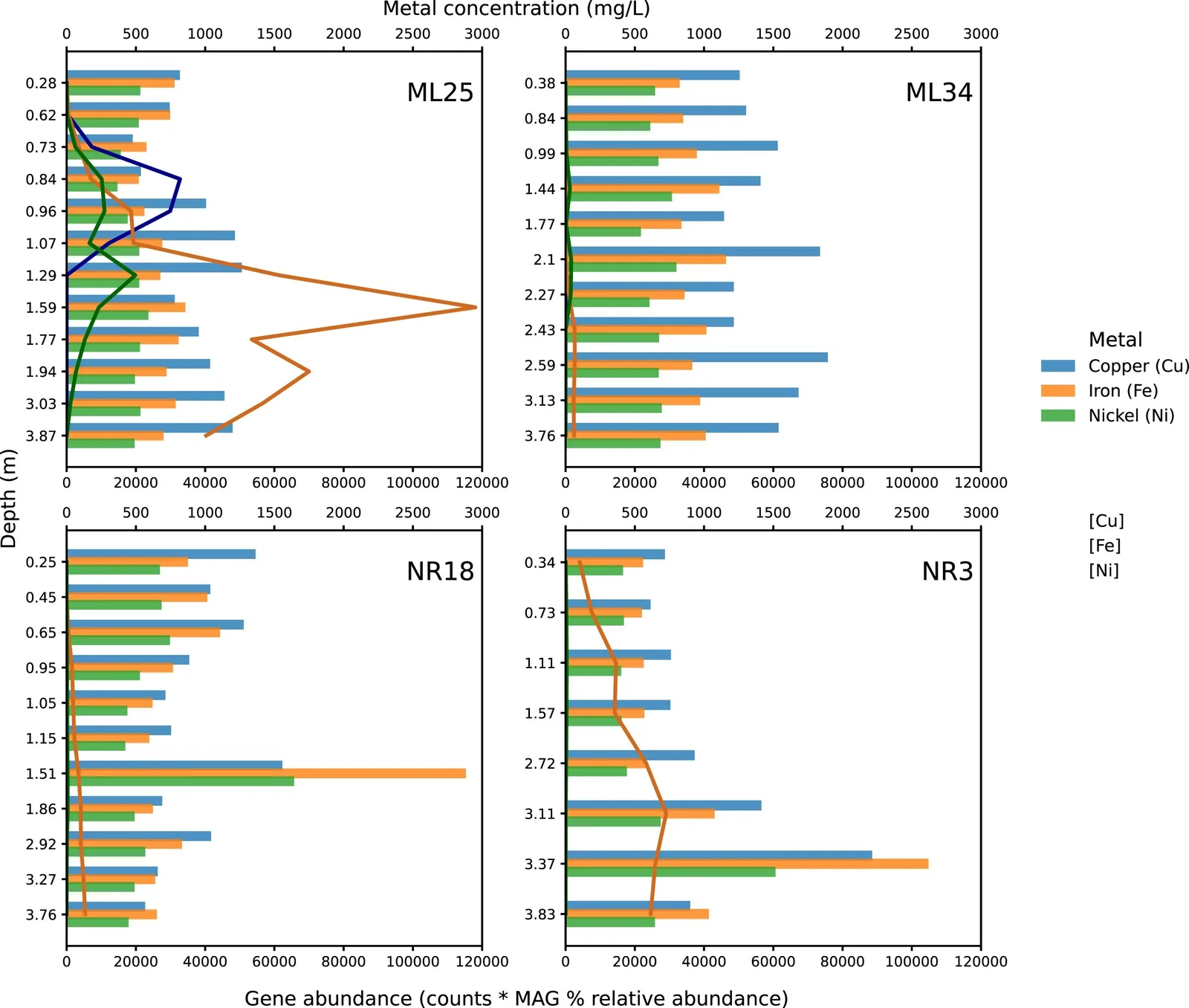
Abundance of metal resistance genes and metal concentrations along vertical mine tailings transects. Bar plots depict relative gene abundances for Cu, Fe, and Ni-associated MRGs. Line plots represent Cu, Fe, and Ni concentrations in tailings pore-water. There was no significant correlation between metal concentrations and associated MRG abundances (p_Cu_ = 0.18, p_Fe_ = 0.79, p_Ni_ = 0.26). Note that the y axes are not scaled proportionally to depth.

There was no significant correlation between individual concentrations of major heavy metals (Cu, Ni, Fe) and their associated predicted MRG abundance (Pearson correlation, p_Cu_ = 0.18, p_Fe_ = 0.79, p_Ni_ = 0.28). More heavily contaminated tailings (*e.g*. ML25) had similar or lower Cu, Ni, and Fe resistance gene abundances to other locations (Fig. 4, Figs. S1–S3). There was also no correlation between metal concentrations and MRG abundances when comparing samples within a core (0.05 ≤ *P* ≤ 0.95), a slight positive correlation between total MRG abundances with pH (r = 0.54, *P* = 0.003), and a slight negative correlation between total MRG abundances with redox potential (r = −0.48, *P* = 0.009), contradictory to the expectation that metals are more bioavailable and mobile at lower pH and higher redox potential, therefore leading to higher MRG abundances. Although higher environmental metal concentrations could select for organisms with more resistance genes targeting those metals, the results from this study suggest that a broad range of predicted MRGs are present in mine tailings microbial communities regardless of the level of heavy metal stress they are currently experiencing. It would be interesting to compare differences in MRGs across a larger scale, to see if the trends of general resistance seen in these deposits, from the same geographic region, hold across broader changes in baseline metal composition (*i.e*. from different mines).

Metal resistance in mine tailings communities could instead be regulated at the gene expression level [62, 63], which cannot be directly inferred from metagenomic sequencing data. In samples with high (>2000) protein counts, resistance genes for most metals were expressed (Fig. 3), confirming that many predicted MRGs are not only encoded in genomes, but also actively expressed by microbes inhabiting tailings environments. Absence in other samples, especially those with few identified proteins, cannot be confidently interpreted as a lack of MRG expression (Fig. 3). Correlations between MRG expression levels and extracellular metal concentrations could not be determined due to the lack of quantitative mass spectrometry data. However, we hypothesize that the same factors influencing MRG abundance in metagenomes, such as the relative toxicity of individual metals and the ability of MRGs to confer multi-metal resistance, are important determinants of protein abundance.

Some MRGs are known to be carried on plasmids [64–66] as opposed to genomic DNA, leading to their potential underrepresentation when only considering MRGs associated with MAGs. We performed an additional analysis to investigate the contribution of MRGs associated with unbinned scaffolds, including mobile genetic elements, relative to the total MRG abundance within the community. We ran the BacMet HMM annotation pipeline again, on all scaffolds >2.5 kbp in length, and hits were mapped to four categories based on the scaffold of origin: binned, unbinned chromosomal, unbinned plasmids, and unbinned phages as defined by PPRmeta [47] (see Methods). When considering the total predicted MRG counts across scaffolds, 40.1% (934 294 out of 2 328 173) were located on unbinned scaffolds, with 14.6% of the total associated with plasmids (Figure S4, Supplemental Data File 1: Table S3). However, when normalizing MRG hits to relative scaffold abundances, only 19.4% of MRGs are associated with unbinned scaffolds (5.9% plasmid) (Figure S4, Supplemental Data File 1: Table S4). These findings support that the majority of MRGs present in the community that could be sequenced and assembled were captured in the binned scaffolds, and that plasmid-associated MRGs may play a relatively minor role in conferring metal resistance in tailings. The fraction of the MRGs encoded on mobile genetic elements did not contain unique MRGs. The most abundant predicted MRGs located on unbinned plasmids (total relative abundance across all samples > 20%) (Supplemental Data File 1: Table S5) were mostly transporters and transporter-associated regulatory genes, which included MRGs targeting tungsten/molybdenum (*tupC, modC, wtpC*), nickel/cobalt (*fecE, nikDE*), copper/silver (*corR, crdR, cusR, copR*), iron (*yfeB, fetA, fbpC, ybtPQ, troB*), and zinc (*znuC, troB, zraR, irlR*). Each of these genes are categorized as chromosomal rather than plasmid-associated in the BacMet database, and were also annotated on binned chromosomal scaffolds in our dataset. If these MRGs are encoded on both chromosomal DNA as well as mobile genetic elements, it is possible that they represent beneficial mechanisms of metal resistance in tailings communities and could be targets for further characterization.

To investigate the metal resistance mechanisms of individual bins, MAGs were ranked by the total number of predicted Cu, Ni, and Fe resistance genes they encoded (Figure S5), as high MRG counts may be indicative of high metal tolerance in an organism. MAGs encoding a high number of copper-resistance genes (top 200 in Cu resistance gene counts) were most commonly members of the Proteobacteria (30.5%), Desulfobacterota (12.5%), Chloroflexota (10.0%), and Acidobacteria (7.5%) (Figure S5). The top 5 MAGs with the highest number of predicted copper-resistance genes were taxonomically novel (Table 3), unclassified at the genus level or above, and may represent interesting targets for future isolation and characterization of copper resistance mechanisms.

**Table 3.** Metagenome-assembled genomes from mine tailings with the top five highest (99^th^ percentile) copper, nickel, and iron resistance gene counts.

Metal	Bin ID	Taxonomic classification	# of genes	Rel. Abundance (%)
Copper	ML34_020_30	f. Myxococcaceae	1 590	0.96
	ML34_015_36	c. Anaerolineae	1 363	0.68
	NR18_007_01	o. Aggregatilineales	1 250	1.80
	ML34_022_13	o. Deferrisomatales	1 222	1.02
	NR3_023_07	f. Pseudopelobacteraceae	1 123	7.38
Nickel	ML34_015_29	*Allorhizobium* sp.	701	0.94
	NR18_015_01	*Bradyrhizobium* sp.	646	100
	NR3_025_02	*Ruminiclostridium* sp.	622	28.87
	NR3_023_12	*Mobilitalea* sp.	608	1.85
	NR18_005_37	o. Acidimicrobiales	594	1.96
Iron	NR3_025_02	*Ruminiclostridium* sp.	1 197	28.88
	ML34_015_29	*Allorhizobium* sp.	1 164	0.94
	NR18_015_01	*Bradyrhizobium* sp.	1 153	100
	NR3_023_12	*Mobilitalea* sp.	1 117	1.85
	NR18_005_37	o. Acidimicrobiales	1 103	1.96

MAGs (Bin ID) are ranked in decreasing order of MRG counts associated with resistance to copper, nickel, or iron (# of genes). The lowest assigned level of GTDB taxonomic classification is reported (c = class, o = order, f = family). MAG relative abundances (Rel. Abundance) refers to the percent relative abundance of a given MAG within their respective sample, calculated based on average scaffold depth of coverage

Compared to Cu resistance, there was more taxonomic overlap between MAGs with high nickel- and high iron-resistance gene counts (Figure S5), as many genes are mapped to both functions in the BacMet database [42]. Most of the top 200 highest nickel- and iron- resistance gene-encoding MAGs were classified to members of the Actinobacteriota (28.3%), Proteobacteria (14.75%), Chloroflexota (13.75%), and Firmicutes (9.23%) (Figure S5). The top five MAGs with highest counts for nickel and for iron-associated MRGs were identical, and included *Allorhizobium* and *Bradyrhizobium* spp. (c. Alphaproteobacteria, o. Rhizobiales) (Table 3). *Bradyrhizobium* and other Rhizobiales spp. are known to be nickel tolerant and promote plant growth in Ni-rich soils, as they are nitrogen-fixing symbionts of legumes [67, 68]. These lineages potentially enhance tailings remediation through Ni uptake and bioaccumulation, thereby decreasing its mobility and impact on other community members [69, 70]. The *Ruminiclostridium* MAG in NR3_025 is artificially abundant, as only two MAGs were binned for that sample. *Ruminiclostridium* may be present due to the presence of organic plant material in deep NR3 samples, as it is expected to use an anaerobic, cellulose degrading metabolism [71].

**Figure 5.**
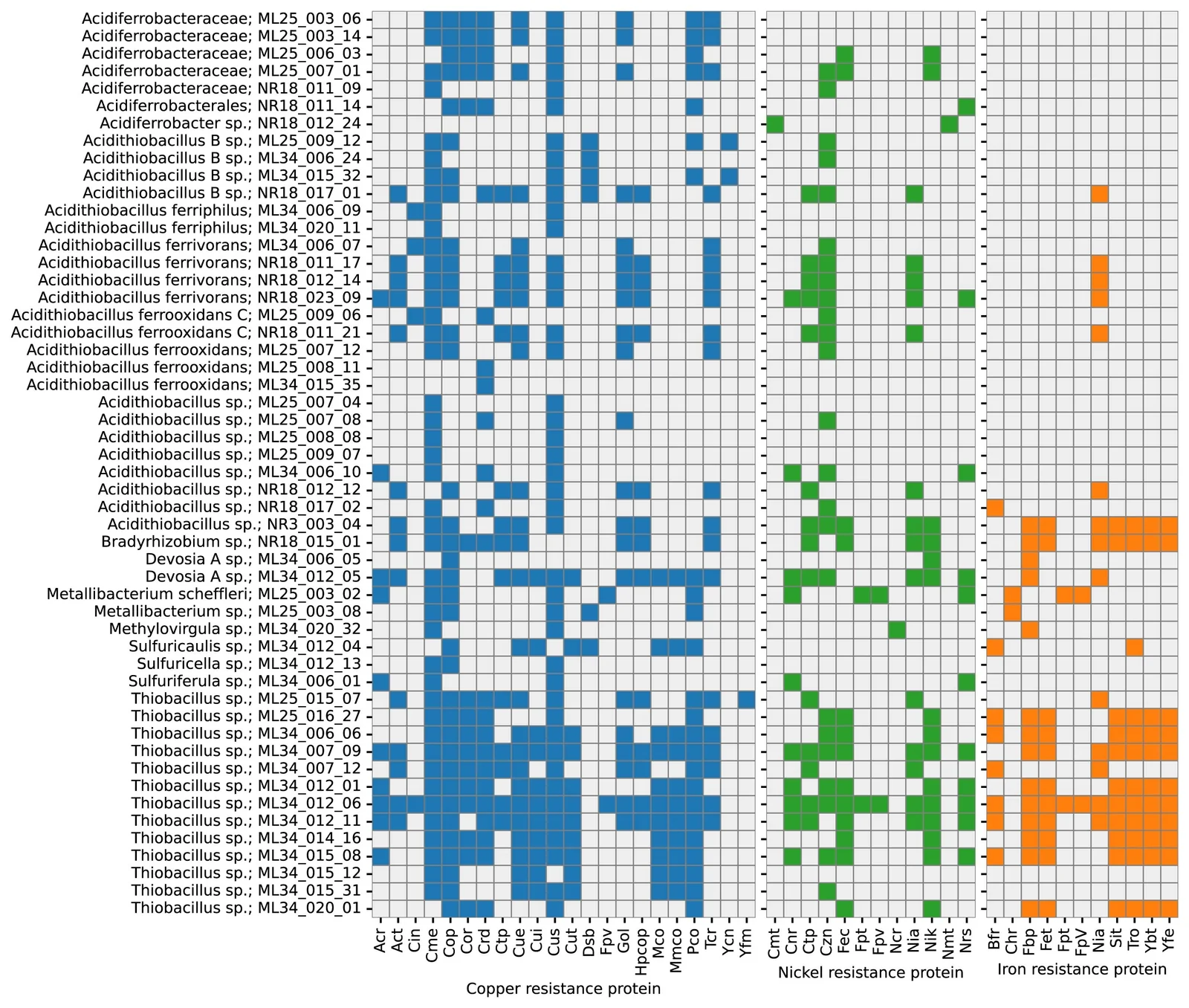
Taxonomic distribution of copper, nickel, and iron resistance proteins in tailings communities. Shaded cells represent positive detection of specific proteins associated with copper, nickel, and iron resistance. Proteins within common pathways (*e.g*. Cop, Cus) were aggregated. Proteins were identified by mass spectrometry and mapped to predicted proteins from metagenomic sequencing, and aggregated by the MAG from which they originated (if any). Bins are labelled by their ID, followed by their lowest level of GTDB-assigned taxonomy. Only bins with >50 unique proteins detected across all samples were included in this analysis.

The MAGs high in Cu, Ni, and Fe resistance genes are predicted to represent heterotrophic organisms (Table 3), giving a diverse pool of potential metal cyclers. However, the majority of detected metal resistance proteins mapped to *Acidithiobacillus, Thiobacillus*, and other autotrophic lineages (Fig. 5), with the exceptions of *Bradyrhizobium, Metallibacterium, Methylovirgula*, and *Devosia* spp., where a much smaller proportion of the predicted metal cyclers were actively expressing these functions. Of these, a *Thiobacillus* spp. from ML34 appeared to express the largest diversity of unique proteins related to copper, nickel, and iron resistance (Fig. 5). Fewer types of metal resistance proteins were detected from the taxa predominantly inhabiting more highly metal-contaminated tailings such as *Acidithiobacillus, Metallibacterium*, and Acidiferrobacteraceae, although specific proteins belonging to MRG complexes such as Cme, Cus, Czn, Nik, and Nia were commonly detected as expressed by these clades.

### Distribution and expression of iron- and sulfur-related genes

**Figure 6.**
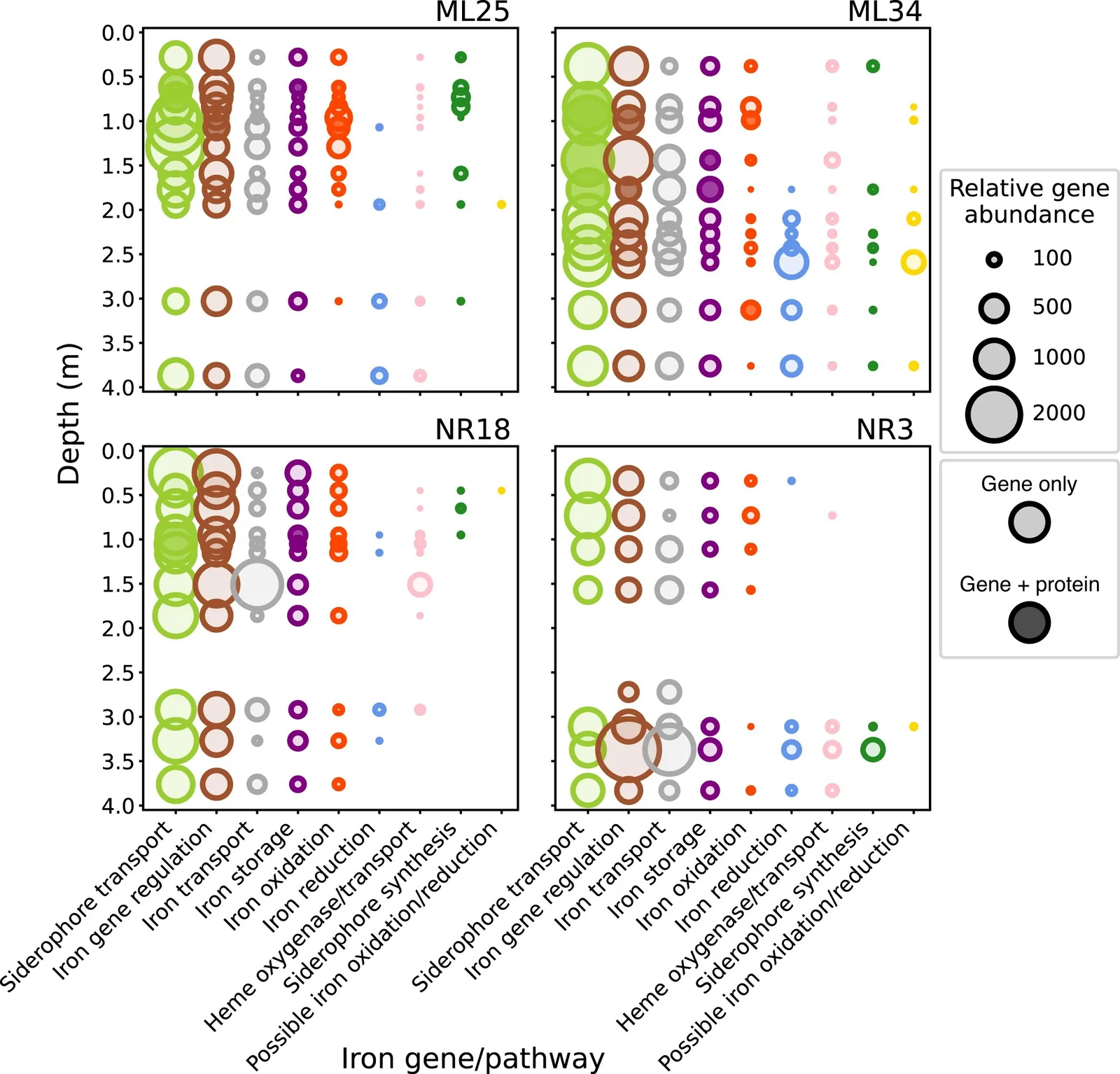
Distribution of iron-related genes across vertical tailings transects. MAGs were annotated using FeGenie. Relative gene abundances were calculated as the number of hits multiplied by the percent relative abundance of MAGs associated with each hit. In samples where protein expression was detected, the corresponding gene(s) are indicated by circles with darker shading. Gene categories are colored based on function (Table S6) [39], ordered from highest overall abundance (Siderophore transport) on the left to lowest (Possible iron oxidation/reduction) on the right.

**Figure 7.**
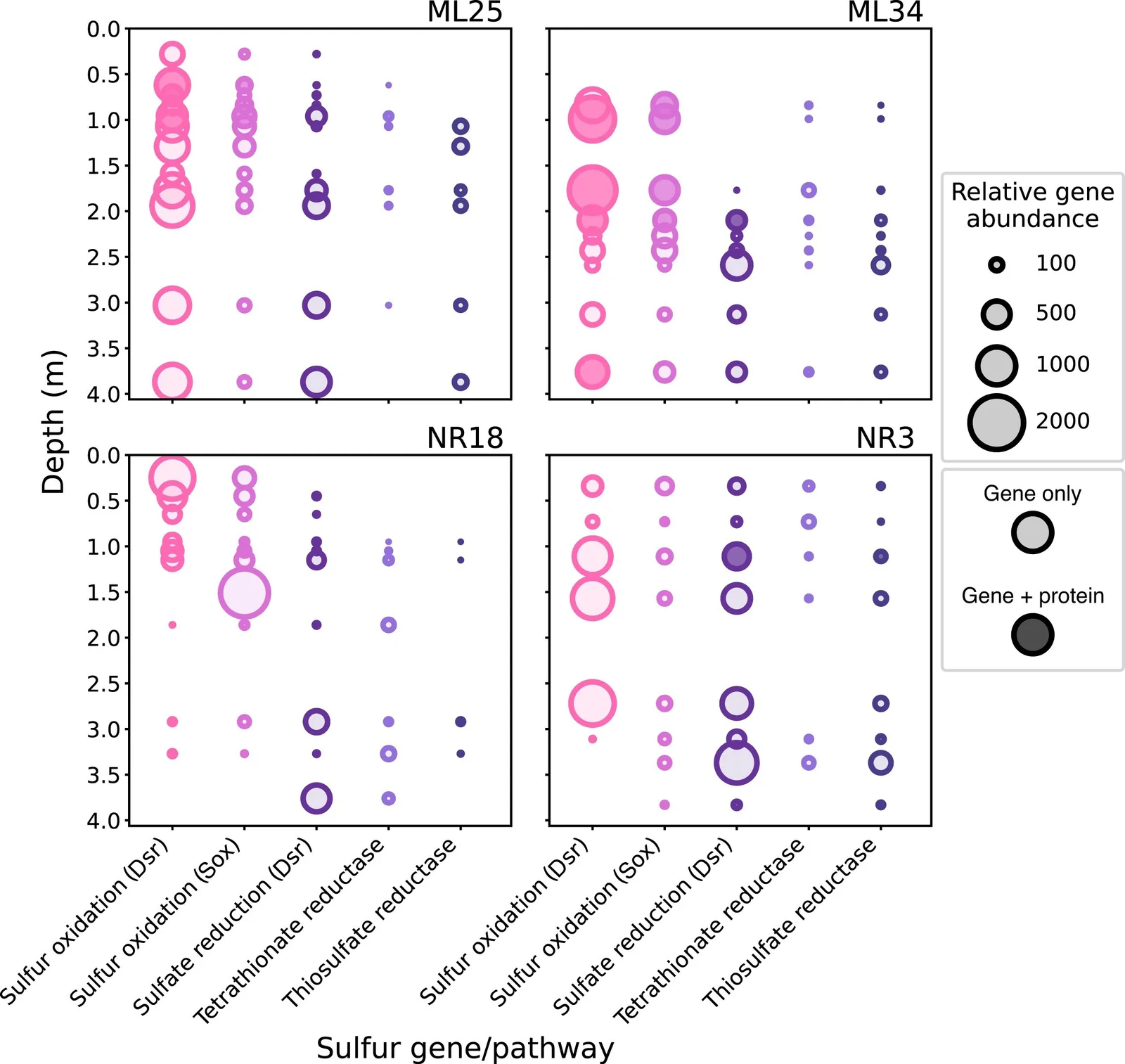
Distribution of sulfur-cycling genes across vertical tailings transects. MAGs were annotated using DRAM (*sox*, tetrathionate reductase, and thiosulfate reductase genes) and DiSCo (*dsr* genes). Relative gene abundances were calculated as the number of hits multiplied by the percent relative abundance of MAGs associated with each hit. In samples where protein expression was detected, the corresponding gene(s) are indicated by circles with darker shading. Gene categories are colored based on function, ordered from highest overall abundance (Sulfur oxidation, Dsr pathway) on the left to lowest (Thiosulfate reductase) on the right.

In general, iron- and sulfur- oxidation genes were more abundant in shallow samples whereas reductive metabolisms were more abundant in deeper samples (Figs. 6 and 7), which was expected based on decreasing oxygen availability and redox potentials (Table S2) with increasing depth. Most predicted iron-oxidizers were bacteria using the *cyc2* pathway [72, 73]. Iron reduction genes were more diverse, being evenly distributed among hits for *dfe_0461-0465, omcF/S/Z*, and other pathways [74, 75]. There was considerable overlap between predictions of iron oxidizing and sulfur oxidizing Gammaproteobacteria, as well as between iron reducing and sulfate reducing Desulfobacterota (Fig. 2). As seen for the MRGs, corresponding proteins were only detected in a few samples (Figs. 6 and 7). In many cases, some genes for a pathway were detected as proteins but not others, and this was inconsistent between samples from similar depths in the same tailings core. Due to this low coverage, protein data does not represent a comprehensive profile of microbial gene expression in these tailings, but provides information as to active processes at the time of sampling.

Differences in potential iron oxidation and *sox*-based sulfur oxidation were most pronounced in ML25, with gene abundances gradually increasing until 1.0–1.5 m, where the microaerophilic environment favored biotic pyrite oxidation, then gradually decreasing as the environment shifted anoxic (Figs. 6 and 7). This trend was mostly attributed to *Acidithiobacillus ferrooxidans* (Fe & S), *Acidiferrobacter* (Fe & S), *Sulfuriferula* (S), and *Sulfuricella* spp. (S), although many other predicted Fe and/or S oxidizers were also present, including *Leptospirillum* (Nitrospirota; Fe), *Metallibacterium* (Proteobacteria; Fe), *Thiomonas* (Proteobacteria; S), unclassified Acidobacteriota MAGs (mainly Fe, some Fe & S), Firmicutes MAGs (varied), *Ca*. Acididesulfobacter (SZUA-79; Fe & S), and *Ca*. Acidulodesulfobacterium spp. (SZUA-79; Fe & S).

The *Ca*. Acidulodesulfobacterales (SZUA-79) MAGs were especially abundant around 1.0 m in depth in ML25 and NR18 (up to 32.6% and 23.0%, respectively). Members of this lineage have been characterized from metagenomic sequencing data from one other AMD environment, with similar hypothesized roles in Fe/S cycling [76], but there is currently no isolated representative for this order. Cyc2 (iron oxidation) protein expression from both *Ca*. Acidulodesulfobacter and *Ca*. Acidulodesulfobacterium was detected (Figure S6), and these Cyc2 proteins were most similar to the Leptospirillaceae Cyc2 variant based on FeGenie HMMs Dissimilatory sulfate reduction activity was also detected from several SZUA-79 MAGs (Figure S7), overlapping imperfectly with Cyc2-expressing MAGs (Figure S6). Iron oxidation and sulfate reduction are not compatible in the same redox environment; proteins from both pathways were detected from each of the SZUA-79 MAGs in the ML25_007 sample (0.73 m), but it is unlikely that sulfate reduction is supported at this depth. However, these results constitute evidence of *Ca*. Acididesulfobacter and *Ca*. Acidulodesulfobacterium being capable of both metabolisms depending on the surrounding geochemical conditions and the availability of different electron acceptors.

Siderophore-related gene abundance followed similar depth patterns as iron oxidation gene abundance in ML25 (Fig. 6), as 95% of predicted iron oxidizers co-encoded siderophore genes. Some siderophore transport proteins were detected, but not proteins for siderophore synthesis, in keeping with geochemical conditions: soluble iron is unlikely to be limiting in tailings environments.

Lower acidity in ML34 tailings supported populations of neutrophilic Fe and/or S oxidizers such as *Thiobacillus, Halothiobacillus*, and *Gallionella* spp. Proteomic data indicate that *Thiobacillus* were using both Dsr and Sox pathways for sulfur oxidation (Figure S7), and that this genus accounted for the majority of iron storage proteins (ferritin) detected (Figure S6). Given that *Thiobacillus* cannot oxidize iron for energy, they most likely use ferritin as an iron resistance mechanism [77] by storing excess iron released from oxidizing pyrite and other minerals containing both iron and reduced inorganic sulfur compounds (RISCs). The expression of FecR, a protein regulating the ferric citrate uptake system, was associated with *Ca*. Devosia spp. in ML34 (Figure S6), which also expressed a variety of Cu, Ni, and Fe-resistance proteins (Fig. 5). Members of this genus are often found in soil environments contaminated with various organic toxins [78]; this study demonstrates their tolerance towards moderate levels of metal contamination as well.

The *Ca*. Eremiobacterota were predicted iron oxidizers unique to NR18 and NR3 samples, and were the most abundant organisms (up to 89%) in several samples. All *Ca*. Eremiobacterota MAGs were classified under *Ca*. Baltobacteraceae, a family whose members are predicted to be metabolically versatile based on previous environmental sequencing and limited cultivation data [79, 80]. Members of the Baltobacteraceae have not previously been identified in mining-associated environments, nor have they previously been predicted to capable of autotrophic iron oxidation [79–82].

The majority (68%) of predicted iron-reducing organisms (mostly from the Desulfobacterota) were identified from ML34 samples below 1.5 m in depth (Fig. 6), which is supported by previous pore-water data on redox potential and oxygen gradients [9]. The previous study consistently showed a shift from oxidizing to reducing environments between 1 and 2 m depths for all cores, alongside a shift in predicted microbial guilds from iron oxidizing to reducing. None of the predicted iron reducers were classified below the family level. No iron reducing gene expression was detected in the proteomics, although this may be due to the fact very few proteins were identified from deeper samples, especially from the ML34 core (Table 2). An abundant (37%) Ignavibacteriaceae (Bacteroidota) MAG in the ML34_020 (3.13 m) sample encoded both iron oxidation and reduction genes, metabolisms which have not been ascribed to the only isolated representative in this genus [83]. Cyc2 expression was detected in this sample (Fig. 6), but the proteins mapped to *Gallionella, Ca*. Acidulodesulfobacterium, and unbinned scaffolds rather than to this Ignavibacteriaceae MAG. The geochemical conditions at 3.13 m depth are unlikely to be supporting significant rates of iron oxidation.

Predicted sulfate-reducing organisms were generally localized to deeper (>2 m) samples at all locations, with some exceptions (such as *dsr* encoded and/or expressed by *Thiobacillus*) (Fig. 7, Figure S7) possibly due to misclassification of oxidative *dsr* genes as reductive. Deeper samples generally had poor binning and protein coverage, and a comprehensive profile of all sulfate reducers cannot be described from these samples. Nonetheless, many novel taxa that were unclassified at the genus level and above were found to both encode and express genes for dissimilatory sulfate reduction. These include members of the Firmicutes (Desulforudaceae, Desulfotomaculales), Desulfobacterota (Desulfobulbaceae, Desulfobaccales, Desulfurivibrionaceae, Desulfomonilaceae), SZUA-79 (discussed above), and one Actinobacteria MAG (Solirubrobacteraceae) (Figure S7). Biological sulfate reduction is often targeted in remediation of heavy metals from mining waste, as heavy metals can be immobilized via sulfide precipitation [8]. Although this method is typically not applicable for remediation of tailings at shallow depths, the presence of sulfate reducing organisms at these depths suggested that the remedial organic carbon cover is effectively removing oxygen, thereby limiting further oxidation of deeper sulfide minerals. The diverse set of novel predicted sulfate reducers identified in these tailings warrant further investigation into their ability to control metal mobility *in situ*, as well as whether measures can be taken to stimulate their activity, such as the addition of organic carbon, nitrogen, and/or phosphorus sources. Improvements in DNA and protein extraction are also required to provide more comprehensive multi-omic analyses of novel sulfate reducers and their capacities for metal  resistance.

A wide variety of taxa appear to be capable of Fe/S cycling in tailings. Vertical heterogeneity in tailings support niches for Fe/S oxidation and reduction, but the exact populations occupying these niches can be highly variable between different cores, and taxonomic composition of these guilds is likely based on selection from environmental factors such as pH and contaminant profiles [9]. Metagenomics enabled the prediction of 301 potential Fe/S cycling MAGs (Fig. 2), of which 139 (46.2%) corresponded to ASVs that could not be confidently linked to these metabolisms based on taxonomic classifications alone [9]. Although most iron- and sulfur-oxidizers identified in this study belong to well-studied genera with cultured representatives (Figure S6, Figure S7), there was considerable species and strain level functional diversity within these groups that potentially influence their roles in heavy metal cycling and/or applicability towards bioleaching [84–87]. Furthermore, organisms encoding and/or expressing proteins for dissimilatory sulfate reduction were largely unclassified at the genus level or above (Figure S7), and were missed in functional predictions from 16S rRNA data [9].

### Conclusions

To our knowledge, this is the first study to apply integrated metagenomic and metaproteomic analyses to mine tailings microbial communities. Metagenomic sequencing revealed a broad range of novel taxa, particularly within the Actinobacteriota, Desulfobacterota, and uncultured phyla such as *Ca*. Eremiobacterota and *Ca*. SZUA-79, some of which were implicated in iron/sulfur cycling processes. Functional profiling revealed distinct ecological strategies employed by differing microbial populations across geochemical gradients. In shallow, oxidized zones, iron- and sulfur-oxidizing Gammaproteobacteria were prevalent and active. Deeper, anoxic layers were dominated by potential sulfate-reducing organisms, many of which were taxonomically novel.

Metal resistance mechanisms within communities as well as in individual taxa were highly diverse, reflecting potential adaptive diversity present within tailings microbiomes. Organisms inhabiting highly contaminated tailings expressed a small set of similar MRGs targeting copper, nickel, and iron, suggesting that microbial adaptations to heavy metal stress more frequently involve the upregulation of a few select pathways for metal detoxification rather than utilization of the broad array of encoded resistance mechanisms observed across many tailings-associated taxa here. The relative toxicity of metals was an important factor influencing MRG abundances in metagenomes, whereas there were no clear correlations between predicted MRG abundances and environmental metal concentrations.

Although proteomics data was sparse and uneven from this challenging matrix, the expression of genes related to metal resistance and iron/sulfur cycling confirmed that identified metal-interacting microbes in these tailings not only encode, but actively utilize, a diverse set of MRGs and MMGs. Functional characterization of these MRGs and other enzymes associated with geochemically-relevant processes in the under-characterize lineages identified here would bolster these predictions, however it is clear from this study that a considerable amount of yet uncharacterized diversity remains within Fe/S cycling consortia found in mine tailings, highlighting the benefit of deeper genomic and functional analyses to understand their ecological roles and evaluate their potential for heavy metal remediation.

## Supplementary Material

mfag028_Supplemental_Files

## Data Availability

All molecular data associated with this project has been made publicly available. Metagenome sequence information is housed on NCBI under BioProject PRJNA1017420. Metagenome reads are available under SRA accessions SRR36271342- SRR36271385. All MAGs are available under accessions JBTRST000000000-JBTSHO000000000. All proteomic data, including working files and raw data are available at the Open Science Framework at https://osf.io/v427n/overview?view_only=dc71d1b6927e4fd18e422b7746e236ab.
